# Supplementary material for: Estimating the Global Spread of Epidemic Human Monkeypox with Bayesian Directed Acyclic Graphic Model
Source: Vaccines (Basel). 2023 Feb 17;11(2):468. doi: 10.3390/vaccines11020468 (PMC9959462; doi:10.3390/vaccines11020468)
Supplement: Supplementary file 1 [file vaccines-11-00468-s001.zip › vaccines-2149705-supplementary.pdf]

Supplementary:

The data on monkeypox outbreaks are listed in the column "Observed cumulated cases" of Table S1 and Table S2. The results of predicted monkeypox cases by the Bayesian SIR model and the deterministic SIR model are listed in Table S1 and Table S2, respectively.

Table S1 The observed cumulated monkeypox cases and predicted cumulated cases by the Bayesian SIR model

(a) The global monkeypox cases

Date	Observed cumulated cases	Predicted cumulated cases	95% CI		Date	Observed cumulated cases	Predicted cumulated cases	95% CI	
2022/1/31	3	1.0	-	-	2022/5/8	37	45.2	32.6	76.4
2022/2/16	3	7.0	6.7	7.7	2022/5/9	37	47.3	34.0	80.3
2022/2/17	6	7.4	7.0	8.2	2022/5/10	37	49.9	35.8	84.8
2022/2/27	6	11.2	10.3	13.1	2022/5/11	37	52.9	37.9	89.9
2022/2/28	7	11.6	10.6	13.6	2022/5/12	38	56.5	40.5	95.8
2022/3/3	7	12.7	11.5	15.2	2022/5/13	39	60.7	43.5	102.6
2022/3/4	9	13.1	11.9	15.7	2022/5/14	39	65.7	47.3	110.3
2022/3/30	9	23.4	19.4	32.2	2022/5/15	43	71.6	51.8	119.2
2022/3/31	15	23.8	19.7	32.9	2022/5/16	43	78.6	57.3	129.5
2022/4/9	15	27.5	22.1	39.9	2022/5/17	46	86.8	63.9	141.2
2022/4/10	19	27.9	22.3	40.7	2022/5/18	67	96.6	71.8	154.7
2022/4/11	19	28.3	22.6	41.6	2022/5/19	86	108.1	81.4	170.1
2022/4/12	21	28.7	22.8	42.4	2022/5/20	131	121.8	93.1	187.9
2022/4/13	21	29.2	23.1	43.2	2022/5/21	147	138.0	107.1	208.3
2022/4/14	21	29.6	23.3	44.1	2022/5/22	147	157.2	124.0	231.7
2022/4/15	21	30.0	23.6	45.0	2022/5/23	220	179.9	144.5	258.6
2022/4/16	21	30.4	23.8	45.8	2022/5/24	256	206.9	169.2	289.4
2022/4/17	21	30.9	24.1	46.7	2022/5/25	300	238.8	199.1	324.9
2022/4/18	21	31.3	24.3	47.6	2022/5/26	388	276.7	235.1	365.5
2022/4/19	21	31.7	24.6	48.5	2022/5/27	440	321.6	278.6	412.2
2022/4/20	21	32.1	24.8	49.4	2022/5/28	457	374.9	331.0	465.8
2022/4/21	21	32.6	25.1	50.4	2022/5/29	477	438.1	395.0	527.8
2022/4/22	21	33.0	25.3	51.3	2022/5/30	600	513.1	471.8	598.2
2022/4/23	21	33.4	25.6	52.2	2022/5/31	665	602.2	564.2	678.8
2022/4/24	21	33.8	25.8	53.2	2022/6/1	749	678.6	641.7	751.9
2022/4/25	21	34.3	26.0	54.1	2022/6/2	874	758.7	722.9	828.5
2022/4/26	21	34.7	26.3	55.1	2022/6/3	968	842.5	808.1	908.9
2022/4/27	21	35.1	26.5	56.1	2022/6/4	976	930.3	897.9	993.8
2022/4/28	21	35.6	26.7	57.1	2022/6/5	1083	1022.2	990.9	1082.0
2022/4/29	21	36.0	27.0	58.1	2022/6/6	1198	1118.5	1089.3	1175.3
2022/4/30	26	36.5	27.2	59.1	2022/6/7	1348	1219.3	1190.6	1271.5
2022/5/1	26	36.9	27.4	60.2	2022/6/8	1445	1324.8	1299.9	1375.4

2022/5/2	26	37.6	27.8	61.6	2022/6/9	1591	1435.3	1411.8	1481.5
2022/5/3	26	38.4	28.3	63.3	2022/6/10	1634	1551.0	1528.1	1592.0
2022/5/4	26	39.3	28.9	65.3	2022/6/11	1643	1672.1	1650.5	1708.5
2022/5/5	26	40.5	29.5	67.5	2022/6/12	1757	1798.9	1778.7	1830.7
2022/5/6	27	41.8	30.4	70.1	2022/6/13	1817	1931.6	1912.9	1959.2
2022/5/7	27	43.3	31.4	73.1	2022/6/14	2061	2070.5	2053.9	2094.1

CI: credible interval

Date	Observed cumulated cases	Predicted cumulated cases	95% CI		Date	Observed cumulated cases	Predicted cumulated cases	95% CI	
2022/6/15	2208	2215.8	2200.9	2235.1	2022/7/23	17016	16773.5	16664.6	16830.9
2022/6/16	2584	2368.0	2354.1	2383.6	2022/7/24	17050	17550.3	17447.8	17606.1
2022/6/17	2778	2527.2	2515.5	2540.4	2022/7/25	18336	18357.5	18261.8	18411.5
2022/6/18	2783	2693.7	2682.6	2705.0	2022/7/26	19872	19196.0	19109.1	19248.5
2022/6/19	2790	2868.1	2856.2	2879.1	2022/7/27	21025	20066.7	19987.4	20116.0
2022/6/20	3084	3050.4	3036.4	3063.2	2022/7/28	21867	20970.4	20900.0	21015.6
2022/6/21	3366	3241.2	3223.6	3256.3	2022/7/29	23087	21908.0	21849.5	21949.4
2022/6/22	3540	3440.7	3419.1	3458.6	2022/7/30	23172	22880.4	22833.5	22917.4
2022/6/23	4098	3649.5	3623.8	3670.2	2022/7/31	23303	23784.5	23746.5	23820.9
2022/6/24	4303	3867.8	3836.1	3890.2	2022/8/1	24521	24688.6	24655.1	24720.5
2022/6/25	4315	4096.0	4058.6	4120.3	2022/8/2	25963	25592.4	25563.8	25623.2
2022/6/26	4511	4334.7	4292.7	4362.1	2022/8/3	26616	26495.5	26467.8	26522.7
2022/6/27	4898	4584.3	4535.1	4612.2	2022/8/4	27740	27397.8	27370.9	27424.3
2022/6/28	5246	4845.2	4792.0	4876.7	2022/8/5	28907	28298.8	28270.2	28324.7
2022/6/29	5399	5117.9	5058.8	5151.2	2022/8/6	29021	29198.3	29167.8	29225.8
2022/6/30	6206	5402.9	5338.6	5438.7	2022/8/7	29193	30096.0	30065.9	30128.9
2022/7/1	6475	5700.7	5631.4	5739.0	2022/8/8	31178	30991.5	30956.0	31025.3
2022/7/2	6519	6011.9	5939.3	6054.4	2022/8/9	32613	31884.5	31848.1	31923.1
2022/7/3	6559	6337.0	6258.9	6381.3	2022/8/10	33766	32774.8	32735.7	32816.2
2022/7/4	7053	6676.6	6593.3	6723.0	2022/8/11	34451	33662.1	33622.9	33708.3
2022/7/5	7500	7031.2	6942.8	7079.2	2022/8/12	35863	34546.0	34504.9	34595.4
2022/7/6	7732	7401.6	7307.9	7451.2	2022/8/13	36004	35426.3	35380.6	35475.3
2022/7/7	8426	7788.2	7690.0	7839.7	2022/8/14	36172	36302.7	36256.9	36355.9
2022/7/8	9486	8191.8	8097.0	8252.5	2022/8/15	37646	37175.0	37127.1	37229.8
2022/7/9	9608	8613.0	8514.7	8675.6	2022/8/16	38965	38042.8	37994.3	38100.1
2022/7/10	9635	9052.5	8944.2	9110.2	2022/8/17	40147	38906.0	38859.8	38968.8
2022/7/11	10226	9511.0	9405.5	9576.2	2022/8/18	41192	39764.2	39717.5	39828.6
2022/7/12	11098	9989.2	9880.2	10055.3	2022/8/19	41964	40617.2	40570.3	40683.2
2022/7/13	11516	10487.8	10372.8	10551.0	2022/8/20	42130	41464.8	41417.4	41531.5
2022/7/14	12127	11007.7	10890.6	11071.4	2022/8/21	42238	42306.7	42259.1	42374.0
2022/7/15	13015	11549.6	11431.3	11614.5	2022/8/22	44301	43142.7	43091.1	43206.7
2022/7/16	13031	12114.2	11996.4	12180.8	2022/8/23	45752	43972.6	43920.8	44036.2
2022/7/17	13069	12702.4	12584.1	12768.7	2022/8/24	46801	44796.1	44744.9	44859.7
2022/7/18	13886	13315.1	13196.6	13381.0	2022/8/25	47438	45613.1	45562.9	45676.6
2022/7/19	15081	13952.9	13834.9	14018.1	2022/8/26	48504	46423.4	46377.8	46489.9
2022/7/20	15655	14616.8	14499.7	14680.4	2022/8/27	48563	47226.8	47176.6	47286.8
2022/7/21	16293	15307.6	15194.3	15371.5	2022/8/28	48616	48023.1	47979.4	48087.4
2022/7/22	17013	16026.2	15912.4	16084.7	2022/8/29	50343	48812.0	48768.3	48873.4

CI: credible interval

Date	Observed cumulated cases	Predicted cumulated cases	95% CI	
2022/8/30	51110	49593.6	49547.9	49649.9
2022/8/31	51982	50367.5	50322.0	50420.1
2022/9/1	52941	51133.8	51090.5	51185.1
2022/9/2	53629	51892.1	51849.2	51939.6
2022/9/3	53678	52642.4	52602.2	52688.4
2022/9/4	53936	53384.6	53345.8	53427.7
2022/9/5	54939	54118.5	54077.4	54155.9
2022/9/6	56138	54844.1	54806.8	54881.3
2022/9/7	56852	55561.2	55524.3	55595.7
2022/9/8	57490	56269.7	56235.5	56304.6
2022/9/9	58211	56969.6	56935.9	57002.6
2022/9/10	58322	57660.9	57628.6	57693.7
2022/9/11	58392	58343.3	58309.4	58374.5
2022/9/12	59351	59017.0	58983.0	59049.9
2022/9/13	60337	59681.7	59646.9	59716.3
2022/9/14	60784	60337.5	60300.3	60375.1
2022/9/15	61581	60984.4	60944.4	61025.7
2022/9/16	62324	61622.2	61578.9	61667.5
2022/9/17	62399	62251.1	62202.8	62300.0
2022/9/18	62403	62870.9	62815.3	62920.6
2022/9/19	63758	63481.7	63420.2	63534.9
2022/9/20	64418	64083.4	64021.6	64145.4
2022/9/21	64904	64676.1	64605.5	64738.6
2022/9/22	65215	65259.8	65182.8	65326.4
2022/9/23	65215	65834.4	65753.8	65907.8
2022/9/24		66400.0	66312.6	66477.1
2022/9/25		66956.7	66861.8	67037.1
2022/9/26		67504.4	67402.0	67588.7
2022/9/27		68043.2	67933.1	68131.6
2022/9/28		68573.2	68456.1	68666.4
2022/9/29		69094.3	68971.4	69193.5
2022/9/30		69606.6	69476.1	69710.3

CI: credible interval

(b) **United States**

Date	Observed cumulated cases	Predicted cumulated cases	95% CI		Date	Observed cumulated cases	Predicted cumulate d cases	95% CI	
2022/5/18	1	1.0			2022/6/25	243	232.0	225.7	247.3
2022/5/19	2	1.6	1.6	1.6	2022/6/26	252	256.0	248.9	273.4
2022/5/20	2	2.3	2.3	2.3	2022/6/27	315	282.5	274.4	302.1
2022/5/21	2	3.0	3.0	3.1	2022/6/28	359	311.6	302.4	333.9
2022/5/22	2	3.8	3.8	3.9	2022/6/29	409	343.7	333.3	368.9
2022/5/23	2	4.7	4.7	4.8	2022/6/30	470	379.0	367.3	407.6
2022/5/24	4	5.7	5.7	5.8	2022/7/1	543	417.9	404.6	450.2
2022/5/25	6	6.8	6.8	6.9	2022/7/2	547	459.1	444.4	494.7
2022/5/26	11	8.0	8.0	8.2	2022/7/3	553	503.7	487.5	542.4
2022/5/27	15	9.4	9.3	9.5	2022/7/4	563	552.0	534.4	593.8
2022/5/28	16	10.8	10.8	11.0	2022/7/5	709	604.3	585.6	649.4
2022/5/29	17	12.4	12.3	12.7	2022/7/6	749	660.9	640.4	708.5
2022/5/30	17	14.2	14.1	14.5	2022/7/7	883	722.1	700.3	772.5
2022/5/31	21	16.2	16.0	16.5	2022/7/8	950	788.4	765.2	841.2
2022/6/1	22	18.3	18.1	18.8	2022/7/9	960	860.2	835.7	915.2
2022/6/2	26	20.7	20.5	21.2	2022/7/10	964	937.9	912.2	994.9
2022/6/3	31	23.3	23.0	23.9	2022/7/11	1058	1021.9	995.0	1080.3
2022/6/4	32	26.2	25.9	26.9	2022/7/12	1122	1112.9	1085.2	1172.7
2022/6/5	32	29.3	29.0	30.2	2022/7/13	1245	1211.3	1182.7	1271.7
2022/6/6	34	32.8	32.4	33.9	2022/7/14	1426	1317.8	1288.4	1378.1
2022/6/7	40	36.6	36.2	37.9	2022/7/15	1767	1433.1	1403.1	1492.8
2022/6/8	45	40.9	40.3	42.3	2022/7/16	1767	1557.7	1527.4	1615.8
2022/6/9	50	45.5	44.9	47.2	2022/7/17	1767	1692.6	1662.2	1748.3
2022/6/10	53	50.7	49.9	52.6	2022/7/18	1995	1838.6	1808.4	1890.8
2022/6/11	55	56.3	55.4	58.6	2022/7/19	2114	1996.4	1968.9	2046.0
2022/6/12	57	62.5	61.5	65.2	2022/7/20	2323	2167.2	2139.6	2209.4
2022/6/13	75	69.4	68.1	72.4	2022/7/21	2503	2351.8	2326.8	2387.1
2022/6/14	83	76.9	75.5	80.4	2022/7/22	2875	2551.5	2528.9	2578.6
2022/6/15	96	85.2	83.6	89.3	2022/7/23	2875	2767.4	2748.0	2786.5
2022/6/16	117	94.4	92.5	99.0	2022/7/24	2875	3000.8	2980.0	3019.1
2022/6/17	128	104.4	102.3	109.8	2022/7/25	3457	3253.0	3217.8	3275.9
2022/6/18	132	115.5	113.0	121.6	2022/7/26	3768	3525.7	3472.5	3555.0
2022/6/19	133	127.8	124.9	134.7	2022/7/27	4630	3820.3	3744.1	3855.4
2022/6/20	137	141.2	138.0	149.2	2022/7/28	4895	4138.7	4034.9	4180.3
2022/6/21	175	156.1	152.3	165.2	2022/7/29	5175	4482.5	4346.4	4531.8
2022/6/22	196	172.4	168.1	182.8	2022/7/30	5175	4853.8	4681.1	4913.4
2022/6/23	218	190.4	185.5	202.2	2022/7/31	5175	5254.7	5041.7	5327.3
2022/6/24	237	210.2	204.7	223.6	2022/8/1	5792	5687.5	5427.4	5774.6

CI: credible interval

Date	Observed cumulated cases	Predicted cumulated cases	95% CI		Date	Observed cumulated cases	Predicted cumulated cases	95% CI	
2022/8/2	6308	6084.3	5796.5	6180.3	2022/9/9	21761	21519.0	21387.3	21871.9
2022/8/3	6599	6484.3	6171.6	6585.8	2022/9/10	21761	21732.8	21646.8	21967.1
2022/8/4	7084	6887.5	6556.6	6995.1	2022/9/11	21761	21934.8	21770.5	22106.9
2022/8/5	7490	7293.8	6951.5	7407.6	2022/9/12	21834	22126.1	21781.5	22247.1
2022/8/6	7491	7703.3	7352.4	7818.8	2022/9/13	22480	22307.2	21803.6	22438.5
2022/8/7	7491	8115.7	7763.5	8233.0	2022/9/14	22619	22478.7	21807.6	22647.5
2022/8/8	8902	8531.2	8182.7	8648.3	2022/9/15	22958	22641.4	21813.0	22853.9
2022/8/9	9461	8949.6	8611.6	9066.0	2022/9/16	23339	22795.6	21820.2	23052.5
2022/8/10	10361	9370.9	9047.6	9483.5	2022/9/17	23339	22941.9	21819.0	23235.6
2022/8/11	10727	9795.1	9496.3	9905.8	2022/9/18	23339	23080.8	21824.4	23419.3
2022/8/12	11131	10222.0	9951.6	10327.6	2022/9/19	23730	23212.6	21820.6	23585.8
2022/8/13	11131	10651.7	10413.1	10747.0	2022/9/20	24041	23337.7	21818.7	23747.6
2022/8/14	11131	11084.0	10881.8	11168.8	2022/9/21	24198	23456.5	21826.9	23911.8
2022/8/15	11844	11518.9	11359.2	11593.8	2022/9/22	24403	23569.4	21827.3	24061.7
2022/8/16	12636	11956.3	11837.8	12018.5	2022/9/23	24403	23676.6	21829.3	24208.1
2022/8/17	13450	12396.3	12314.2	12445.6	2022/9/9	21761	21519.0	21387.3	21871.9
2022/8/18	14049	12838.6	12775.6	12896.5	2022/9/10	21761	21732.8	21646.8	21967.1
2022/8/19	14049	13283.2	13219.4	13402.9	2022/9/11	21761	21934.8	21770.5	22106.9
2022/8/20	14049	13730.1	13658.4	13933.0	2022/9/12	21834	22126.1	21781.5	22247.1
2022/8/21	14049	14179.2	14085.8	14471.7	2022/9/13	22480	22307.2	21803.6	22438.5
2022/8/22	15357	14630.5	14516.5	15020.6	2022/9/14	22619	22478.7	21807.6	22647.5
2022/8/23	15832	15083.7	14943.5	15577.3	2022/9/15	22958	22641.4	21813.0	22853.9
2022/8/24	16514	15538.9	15373.9	16146.8	2022/9/16	23339	22795.6	21820.2	23052.5
2022/8/25	16837	15996.0	15808.0	16730.9	2022/9/17	23339	22941.9	21819.0	23235.6
2022/8/26	17336	16454.9	16231.9	17312.6	2022/9/18	23339	23080.8	21824.4	23419.3
2022/8/27	17336	16915.4	16659.7	17910.6	2022/9/19	23730	23212.6	21820.6	23585.8
2022/8/28	17336	17377.7	17087.2	18519.3	2022/9/20	24041	23337.7	21818.7	23747.6
2022/8/29	17986	17841.4	17511.9	19133.9	2022/9/21	24198	23456.5	21826.9	23911.8
2022/8/30	18298	18306.6	17940.3	19762.6	2022/9/22	24403	23569.4	21827.3	24061.7
2022/8/31	18879	18773.1	18361.8	20394.9	2022/9/23	24403	23676.6	21829.3	24208.1
2022/9/1	19355	19241.0	18791.5	21046.3	2022/9/24		23778.4	21813.6	24329.9
2022/9/2	19852	19597.6	19172.1	21254.8	2022/9/25		23875.1	21813.1	24459.4
2022/9/3	19852	19928.9	19540.7	21409.1	2022/9/26		23967.0	21813.7	24584.5
2022/9/4	19852	20237.8	19888.4	21516.7	2022/9/27		24054.4	21802.1	24690.6
2022/9/5	19852	20526.8	20219.1	21596.5	2022/9/28		24137.4	21802.2	24803.2
2022/9/6	20608	20797.9	20536.7	21660.8	2022/9/29		24216.2	21800.9	24909.3
2022/9/7	21148	21052.7	20828.6	21714.2	2022/9/30		24291.2	21802.2	25012.1
2022/9/8	21374	21292.6	21113.3	21781.1					

CI: credible interval

(c) **Spain**

Date	Observed cumulated cases	Predicted cumulated cases	95% CI		Date	Observed cumulated cases	Predicted cumulated cases	95% CI	
2022/5/18	7	7.0			2022/6/25	736	718.7	708.3	727.9
2022/5/19	7	10.5	10.4758	10.5114	2022/6/26	736	767.9	757	777.6
2022/5/20	30	14.3	14.2303	14.3042	2022/6/27	800	819.7	808.6	830.1
2022/5/21	40	18.3	18.2872	18.4028	2022/6/28	800	874.2	862.8	884.9
2022/5/22	40	22.8	22.6646	22.8257	2022/6/29	800	931.5	919.7	942.6
2022/5/23	41	27.5	27.3974	27.6092	2022/6/30	1196	991.7	979.6	1003.2
2022/5/24	45	32.6	32.506	32.7768	2022/7/1	1196	1055.1	1043	1067.4
2022/5/25	53	38.2	38.022	38.3584	2022/7/2	1196	1121.8	1109.4	1134.2
2022/5/26	78	44.2	43.9787	44.3912	2022/7/3	1196	1191.9	1179.5	1204.7
2022/5/27	99	50.7	50.3905	50.8949	2022/7/4	1258	1265.6	1253.5	1279
2022/5/28	99	57.7	57.3301	57.9349	2022/7/5	1258	1343.2	1330.6	1356.4
2022/5/29	108	65.2	64.8154	65.5441	2022/7/6	1258	1424.7	1411.9	1437.7
2022/5/30	116	73.4	72.9079	73.7746	2022/7/7	1258	1510.5	1497.9	1523.8
2022/5/31	136	82.2	81.6215	82.6531	2022/7/8	2034	1600.7	1587.9	1613.9
2022/6/1	136	91.7	91.0529	92.2739	2022/7/9	2034	1695.5	1682.9	1708.9
2022/6/2	162	101.9	101.2	102.7	2022/7/10	2034	1795.2	1782.7	1808.7
2022/6/3	181	113.0	112.2	113.9	2022/7/11	2034	1900.0	1886.9	1912.6
2022/6/4	181	125.0	124	126	2022/7/12	2447	2010.2	1998.2	2023.9
2022/6/5	181	138.0	136.8	139.1	2022/7/13	2447	2126.0	2113.4	2139
2022/6/6	198	151.9	150.6	153.3	2022/7/14	2447	2247.8	2234.6	2260.7
2022/6/7	225	167.0	165.5	168.6	2022/7/15	2835	2375.8	2362.7	2389.5
2022/6/8	259	183.3	181.6	185.1	2022/7/16	2835	2510.3	2496.8	2524.5
2022/6/9	259	200.9	198.9	203	2022/7/17	2835	2651.7	2637	2666.4
2022/6/10	275	219.9	217.6	222.3	2022/7/18	2835	2774.6	2760.3	2790.9
2022/6/11	275	240.5	237.8	243.1	2022/7/19	3125	2895.7	2880.2	2912
2022/6/12	275	262.6	259.6	265.7	2022/7/20	3125	3014.8	2998.1	3030.9
2022/6/13	275	286.6	283.1	290.1	2022/7/21	3125	3132.0	3115	3149
2022/6/14	313	312.4	308.5	316.4	2022/7/22	3126	3247.3	3230	3265.3
2022/6/15	313	340.3	335.8	344.8	2022/7/23	3126	3360.8	3342.7	3379
2022/6/16	497	370.5	365.3	375.4	2022/7/24	3126	3472.5	3453.6	3490.7
2022/6/17	498	403.0	397.1	408.6	2022/7/25	3126	3582.4	3562.8	3600.8
2022/6/18	498	435.9	429.4	442	2022/7/26	3738	3690.5	3670.7	3709.1
2022/6/19	498	470.4	463.3	477	2022/7/27	3738	3796.9	3777.1	3816
2022/6/20	521	506.7	499.1	513.8	2022/7/28	3738	3901.5	3881.8	3920.9
2022/6/21	521	544.9	536.7	552.5	2022/7/29	4299	4004.5	3984.5	4023.9
2022/6/22	521	585.1	576.4	593.1	2022/7/30	4300	4105.7	4085.4	4124.9
2022/6/23	736	627.4	618.1	635.9	2022/7/31	4300	4205.4	4185.4	4224.8
2022/6/24	736	671.9	662	680.8	2022/8/1	4300	4303.4	4283.1	4322.4

CI: credible interval

Date	Observed cumulated cases	Predicted cumulated cases	95% CI		Date	Observed cumulated cases	Predicted cumulated cases	95% CI	
2022/8/2	4577	4399.7	4379.8	4418.8	2022/9/9	6884	6887.5	6865.8	6909.5
2022/8/3	4577	4494.6	4475	4513.8	2022/9/10	6884	6905.3	6883.4	6927.3
2022/8/4	4577	4587.8	4568.6	4607.1	2022/9/11	6884	6921.2	6899.1	6944.4
2022/8/5	4942	4679.5	4660.6	4698.6	2022/9/12	6884	6935.6	6912.9	6960.4
2022/8/6	4942	4769.7	4751.1	4788.8	2022/9/13	6947	6948.5	6924.7	6975
2022/8/7	4942	4858.4	4840.5	4877.8	2022/9/14	6947	6960.1	6933.9	6987.3
2022/8/8	4942	4945.7	4927.7	4964.5	2022/9/15	6947	6970.5	6942.4	6999.2
2022/8/9	5162	5031.4	5014.3	5050.3	2022/9/16	7037	6979.9	6950.7	7010.9
2022/8/10	5162	5115.8	5098.7	5134.4	2022/9/17	7037	6988.3	6957.3	7021.3
2022/8/11	5162	5198.8	5181.5	5216.7	2022/9/18	7037	6995.9	6962.1	7029.8
2022/8/12	5719	5280.3	5262.7	5297.7	2022/9/19	7037	7002.8	6967.9	7039.4
2022/8/13	5719	5360.5	5343.6	5378.3	2022/9/20	7083	7008.9	6971.7	7046.6
2022/8/14	5719	5439.4	5422.2	5457.1	2022/9/21	7083	7014.4	6975.8	7054.1
2022/8/15	5719	5516.9	5499.8	5534.8	2022/9/22	7083	7019.4	6978.4	7060.1
2022/8/16	5792	5593.2	5575.4	5611	2022/9/23	7083	7023.9	6981.6	7066.4
2022/8/17	5792	5668.1	5650.1	5686.3	2022/9/9	6884	7027.9	6984.7	7072.5
2022/8/18	5792	5741.8	5723.8	5761	2022/9/10	6884	7031.6	6986.8	7077.7
2022/8/19	6119	5814.2	5795.7	5834.1	2022/9/11	6884	7034.8	6988.6	7082.3
2022/8/20	6119	5885.4	5866.1	5905.5	2022/9/12	6884	7037.8	6990.5	7086.9
2022/8/21	6119	5955.4	5935.3	5976.1	2022/9/13	6947	7040.4	6992	7091
2022/8/22	6119	6024.3	6002.3	6045	2022/9/14	6947	7042.8	6990.2	7091.4
2022/8/23	6284	6091.9	6071.1	6115.6	2022/9/15	6947	7045.0	6994.9	7098.4
2022/8/24	6284	6158.4	6134.9	6181.4	2022/9/16	7037	6887.5	6865.8	6909.5
2022/8/25	6284	6223.8	6199.2	6247.9	2022/9/17	7037	6905.3	6883.4	6927.3
2022/8/26	6459	6288.0	6262.9	6314	2022/9/18	7037	6921.2	6899.1	6944.4
2022/8/27	6459	6351.2	6324.7	6378.3	2022/9/19	7037	6935.6	6912.9	6960.4
2022/8/28	6459	6413.2	6386	6442.2	2022/9/20	7083	6948.5	6924.7	6975
2022/8/29	6459	6474.2	6446.3	6505.2	2022/9/21	7083	6960.1	6933.9	6987.3
2022/8/30	6543	6534.2	6505.3	6567.2	2022/9/22	7083	6970.5	6942.4	6999.2
2022/8/31	6543	6593.1	6561.9	6626.7	2022/9/23	7083	6979.9	6950.7	7010.9
2022/9/1	6543	6651.0	6618.8	6686.3	2022/9/24		6988.3	6957.3	7021.3
2022/9/2	6645	6692.7	6662.5	6727.2	2022/9/25		6995.9	6962.1	7029.8
2022/9/3	6645	6730.2	6699	6760.5	2022/9/26		7002.8	6967.9	7039.4
2022/9/4	6645	6763.9	6735.4	6792.6	2022/9/27		7008.9	6971.7	7046.6
2022/9/5	6645	6794.2	6765.8	6819	2022/9/28		7014.4	6975.8	7054.1
2022/9/6	6749	6821.4	6796.7	6846.1	2022/9/29		7019.4	6978.4	7060.1
2022/9/7	6749	6845.8	6822.4	6869.2	2022/9/30		7023.9	6981.6	7066.4
2022/9/8	6749	6867.8	6846.1	6890.9					

CI: credible interval

(d) **Brazil**

Date	Observed cumulated cases	Predicted cumulated cases	95% CI		Date	Observed cumulated cases	Predicted cumulated cases	95% CI	
2022/6/8	1	1.0			2022/7/16	347	355.8	349.0	362.6
2022/6/9	1	1.5	1.5	1.5	2022/7/17	347	388.8	381.6	396.0
2022/6/10	1	2.1	2.1	2.1	2022/7/18	347	424.2	416.5	431.8
2022/6/11	2	2.8	2.8	2.8	2022/7/19	448	462.1	454.1	470.2
2022/6/12	3	3.5	3.5	3.6	2022/7/20	591	502.8	494.4	511.4
2022/6/13	3	4.4	4.4	4.4	2022/7/21	604	546.5	537.7	555.5
2022/6/14	5	5.4	5.3	5.4	2022/7/22	694	593.4	584.2	602.8
2022/6/15	5	6.5	6.4	6.5	2022/7/23	694	643.6	633.7	653.0
2022/6/16	6	7.7	7.7	7.8	2022/7/24	694	697.6	687.5	707.6
2022/6/17	7	9.1	9.0	9.2	2022/7/25	809	755.4	744.5	765.2
2022/6/18	7	10.7	10.6	10.8	2022/7/26	865	817.4	806.3	827.4
2022/6/19	8	12.5	12.4	12.6	2022/7/27	977	884.0	873.5	895.1
2022/6/20	8	14.6	14.4	14.7	2022/7/28	1066	955.3	945.1	967.0
2022/6/21	9	16.9	16.7	17.0	2022/7/29	1259	1031.9	1021.5	1043.7
2022/6/22	11	19.5	19.3	19.7	2022/7/30	1343	1113.9	1103.6	1125.9
2022/6/23	16	22.5	22.2	22.7	2022/7/31	1370	1202.0	1190.4	1212.7
2022/6/24	17	25.8	25.6	26.1	2022/8/1	1475	1296.4	1285.5	1307.6
2022/6/25	19	29.6	29.3	30.0	2022/8/2	1603	1397.6	1386.0	1408.4
2022/6/26	20	34.0	33.6	34.4	2022/8/3	1721	1506.1	1495.0	1517.7
2022/6/27	20	38.8	38.4	39.4	2022/8/4	1860	1622.5	1611.4	1634.2
2022/6/28	21	44.4	43.8	45.0	2022/8/5	2004	1747.2	1735.5	1759.0
2022/6/29	37	50.6	50.0	51.4	2022/8/6	2108	1881.0	1868.3	1893.1
2022/6/30	49	57.7	56.9	58.6	2022/8/7	2131	2024.4	2011.8	2038.2
2022/7/1	64	65.7	64.8	66.8	2022/8/8	2293	2147.6	2134.5	2162.1
2022/7/2	76	74.8	73.7	76.1	2022/8/9	2415	2270.4	2255.7	2284.6
2022/7/3	78	85.1	83.7	86.6	2022/8/10	2458	2392.7	2377.2	2407.5
2022/7/4	80	96.8	95.1	98.5	2022/8/11	2458	2514.5	2498.8	2530.5
2022/7/5	106	110.0	108.1	112.1	2022/8/12	2746	2635.9	2619.3	2652.2
2022/7/6	142	124.9	122.6	127.4	2022/8/13	2848	2756.8	2739.5	2773.6
2022/7/7	172	141.9	139.1	144.8	2022/8/14	2893	2877.2	2859.0	2893.9
2022/7/8	204	161.0	157.8	164.5	2022/8/15	2985	2997.2	2979.0	3014.9
2022/7/9	218	179.8	176.2	183.7	2022/8/16	3183	3116.7	3097.7	3134.5
2022/7/10	218	200.0	195.9	204.3	2022/8/17	3359	3235.7	3216.5	3254.0
2022/7/11	228	221.6	217.1	226.3	2022/8/18	3450	3354.2	3335.6	3373.6
2022/7/12	266	244.8	239.8	249.9	2022/8/19	3655	3472.2	3453.4	3491.8
2022/7/13	308	269.7	264.2	275.2	2022/8/20	3755	3589.8	3570.6	3609.3
2022/7/14	347	296.4	290.6	302.5	2022/8/21	3787	3706.8	3687.4	3726.4
2022/7/15	347	325.1	318.7	331.4	2022/8/22	3896	3823.4	3804.3	3843.3

Date	Observed cumulated cases	Predicted cumulated cases	95% CI		Date	Observed cumulated cases	Predicted cumulated cases	95% CI	
2022/8/23	3984	3939.4	3919.5	3958.7	2022/9/30		7959.5	7899.1	8017.8
2022/8/24	4144	4054.9	4035.1	4074.2					
2022/8/25	4216	4170.0	4151.2	4190.1					
2022/8/26	4472	4284.5	4264.7	4303.4					
2022/8/27	4472	4398.5	4379.5	4417.9					
2022/8/28	4472	4512.0	4493.2	4531.1					
2022/8/29	4692	4625.0	4606.8	4644.4					
2022/8/30	4876	4737.5	4719.1	4756.7					
2022/8/31	5037	4849.5	4830.9	4868.2					
2022/9/1	5197	4960.9	4942.2	4979.3					
2022/9/2	5197	5071.8	5053.3	5090.4					
2022/9/3	5197	5182.2	5162.9	5200.0					
2022/9/4	5409	5292.1	5273.1	5310.0					
2022/9/5	5525	5401.4	5382.6	5419.3					
2022/9/6	5692	5510.2	5491.3	5528.6					
2022/9/7	5726	5618.5	5599.2	5637.1					
2022/9/8	5852	5726.3	5705.6	5744.6					
2022/9/9	5971	5833.5	5812.3	5852.5					
2022/9/10	6014	5940.1	5919.1	5960.9					
2022/9/11	6032	6046.3	6025.0	6068.4					
2022/9/12	6129	6151.9	6129.8	6175.0					
2022/9/13	6246	6256.9	6232.1	6279.1					
2022/9/14	6448	6361.5	6336.1	6385.4					
2022/9/15	6649	6465.4	6439.5	6491.8					
2022/9/16	6806	6568.9	6540.8	6596.2					
2022/9/17	6867	6671.7	6642.9	6701.6					
2022/9/18	6867	6774.1	6742.5	6804.9					
2022/9/19	7018	6875.9	6843.0	6908.8					
2022/9/20	7115	6977.1	6942.5	7012.5					
2022/9/21	7205	7077.8	7040.5	7114.6					
2022/9/22	7300	7178.0	7138.7	7217.2					
2022/9/23	7300	7277.6	7236.2	7318.8					
2022/9/24		7376.7	7332.5	7419.5					
2022/9/25		7475.2	7428.2	7520.2					
2022/9/26		7573.1	7523.4	7620.6					
2022/9/27		7670.5	7617.7	7719.9					
2022/9/28		7767.4	7712.1	7820.1					
2022/9/29		7863.7	7806.0	7919.2					

(e) **United Kingdom**

Date	Observed cumulated cases	Predicted cumulated cases	95% CI		Date	Observed cumulated cases	Predicted cumulated cases	95% CI	
2022/5/6	1	1			2022/6/13	470	446.7	439.0	454.2
2022/5/7	1	1.6	1.6	1.6	2022/6/14	524	478.2	470.0	485.9
2022/5/8	1	2.2	2.2	2.3	2022/6/15	524	510.5	502.1	518.5
2022/5/9	1	3.0	3.0	3.0	2022/6/16	574	543.8	535.5	552.5
2022/5/10	1	3.9	3.9	3.9	2022/6/17	574	578.1	569.5	586.9
2022/5/11	1	4.9	4.8	4.9	2022/6/18	574	613.4	604.5	622.3
2022/5/12	2	6.0	6.0	6.0	2022/6/19	574	649.6	640.5	658.6
2022/5/13	3	7.3	7.3	7.3	2022/6/20	793	687.0	677.9	696.1
2022/5/14	3	8.8	8.7	8.8	2022/6/21	793	725.4	716.1	734.5
2022/5/15	7	10.5	10.4	10.6	2022/6/22	793	764.9	755.6	774.0
2022/5/16	7	12.4	12.4	12.5	2022/6/23	910	805.5	796.1	814.7
2022/5/17	7	14.7	14.6	14.8	2022/6/24	910	847.3	837.9	856.5
2022/5/18	9	17.2	17.1	17.3	2022/6/25	910	890.3	881.6	900.2
2022/5/19	9	20.1	20.0	20.3	2022/6/26	1076	934.6	925.9	944.4
2022/5/20	20	23.5	23.3	23.7	2022/6/27	1076	980.1	971.5	989.9
2022/5/21	20	27.3	27.0	27.5	2022/6/28	1076	1027.0	1018.2	1036.5
2022/5/22	20	31.7	31.4	32.0	2022/6/29	1076	1075.1	1066.2	1084.4
2022/5/23	57	36.6	36.3	37.0	2022/6/30	1235	1124.7	1116.0	1134.2
2022/5/24	71	42.4	41.9	42.8	2022/7/1	1235	1175.7	1166.4	1184.8
2022/5/25	78	48.9	48.3	49.5	2022/7/2	1235	1228.1	1218.1	1237.0
2022/5/26	106	56.4	55.7	57.1	2022/7/3	1235	1282.0	1272.0	1291.3
2022/5/27	106	64.9	64.1	65.7	2022/7/4	1351	1337.5	1327.4	1347.6
2022/5/28	106	74.7	73.7	75.7	2022/7/5	1351	1394.5	1384.2	1405.4
2022/5/29	106	85.9	84.6	87.1	2022/7/6	1351	1446.6	1435.9	1457.9
2022/5/30	179	98.7	97.2	100.1	2022/7/7	1552	1497.9	1486.8	1509.7
2022/5/31	190	113.3	111.5	115.0	2022/7/8	1552	1548.5	1537.0	1560.7
2022/6/1	196	130.1	127.8	132.1	2022/7/9	1552	1598.5	1586.3	1610.8
2022/6/2	207	149.3	146.7	151.7	2022/7/10	1552	1647.8	1635.8	1661.0
2022/6/3	226	171.2	168.0	174.1	2022/7/11	1735	1696.4	1683.8	1709.8
2022/6/4	226	196.3	192.5	199.8	2022/7/12	1735	1744.4	1731.0	1757.6
2022/6/5	226	224.9	220.4	229.1	2022/7/13	1735	1791.8	1778.3	1805.3
2022/6/6	302	250.0	245.1	254.7	2022/7/14	1856	1838.5	1824.9	1852.4
2022/6/7	321	275.7	270.3	280.8	2022/7/15	1856	1884.6	1870.7	1898.6
2022/6/8	321	302.2	296.4	307.8	2022/7/16	1856	1930.1	1916.0	1944.2
2022/6/9	366	329.5	323.2	335.5	2022/7/17	1856	1975.0	1960.8	1989.4
2022/6/10	366	357.5	350.9	364.0	2022/7/18	2137	2019.2	2005.0	2033.7
2022/6/11	366	386.4	379.4	393.2	2022/7/19	2137	2062.9	2048.5	2077.2
2022/6/12	470	416.1	408.8	423.3	2022/7/20	2137	2106.0	2091.4	2120.2

Date	Observed cumulated cases	Predicted cumulated cases	95% CI		Date	Observed cumulated cases	Predicted cumulated cases	95% CI	
2022/7/21	2208	2148.5	2134.3	2163.2	2022/8/28	3340	3397.5	3366.5	3419.0
2022/7/22	2208	2190.4	2176.5	2205.4	2022/8/29	3413	3422.2	3392.1	3446.6
2022/7/23	2208	2231.8	2218.1	2246.8	2022/8/30	3413	3446.7	3414.2	3470.8
2022/7/24	2208	2272.6	2258.8	2287.3	2022/8/31	3413	3470.7	3437.9	3496.5
2022/7/25	2497	2312.8	2299.2	2327.7	2022/9/1	3413	3494.5	3460.5	3521.5
2022/7/26	2497	2352.5	2338.9	2367.2	2022/9/2	3413	3504.1	3472.8	3526.6
2022/7/27	2497	2391.7	2378.0	2405.9	2022/9/3	3413	3511.9	3485.6	3532.2
2022/7/28	2546	2430.3	2417.0	2444.7	2022/9/4	3413	3518.3	3496.6	3537.1
2022/7/29	2546	2468.4	2455.2	2482.6	2022/9/5	3484	3523.4	3504.0	3540.3
2022/7/30	2546	2506.0	2492.5	2519.5	2022/9/6	3484	3527.6	3509.7	3543.8
2022/7/31	2546	2543.1	2530.3	2557.1	2022/9/7	3484	3531.0	3514.8	3548.8
2022/8/1	2759	2579.6	2566.0	2592.5	2022/9/8	3484	3533.8	3516.9	3552.4
2022/8/2	2759	2615.7	2602.4	2628.6	2022/9/9	3484	3536.1	3518.3	3556.0
2022/8/3	2759	2651.3	2638.8	2664.9	2022/9/10	3484	3538.0	3518.0	3558.5
2022/8/4	2859	2686.4	2673.2	2699.2	2022/9/11	3484	3539.6	3519.9	3563.8
2022/8/5	2859	2721.0	2707.9	2733.6	2022/9/12	3552	3540.9	3516.2	3563.0
2022/8/6	2859	2755.1	2741.9	2767.6	2022/9/13	3552	3542.0	3517.8	3567.6
2022/8/7	2859	2788.8	2775.6	2801.5	2022/9/14	3552	3542.9	3517.0	3569.4
2022/8/8	3017	2822.0	2808.7	2834.7	2022/9/15	3552	3543.7	3517.2	3572.1
2022/8/9	3017	2854.7	2841.1	2867.3	2022/9/16	3552	3544.3	3516.9	3574.1
2022/8/10	3017	2887.0	2873.0	2900.0	2022/9/17	3552	3544.9	3517.7	3576.9
2022/8/11	3017	2918.8	2904.3	2932.1	2022/9/18	3552	3545.3	3517.9	3578.8
2022/8/12	3017	2950.2	2934.9	2963.6	2022/9/19	3552	3545.7	3517.3	3579.8
2022/8/13	3017	2981.2	2965.7	2995.2	2022/9/20	3585	3546.0	3518.0	3581.9
2022/8/14	3017	3011.8	2996.1	3026.4	2022/9/21	3585	3546.3	3518.1	3583.1
2022/8/15	3195	3041.9	3025.5	3056.9	2022/9/22	3585	3546.5	3518.5	3584.5
2022/8/16	3195	3071.6	3054.5	3087.1	2022/9/23	3585	3546.7	3518.5	3585.5
2022/8/17	3195	3100.9	3082.3	3115.9	2022/9/24		3546.9	3518.2	3586.1
2022/8/18	3195	3129.8	3111.7	3146.8	2022/9/25		3547.1	3518.2	3586.9
2022/8/19	3195	3158.3	3138.3	3174.7	2022/9/26		3547.2	3517.4	3586.7
2022/8/20	3195	3186.4	3165.9	3203.6	2022/9/27		3547.3	3517.4	3587.3
2022/8/21	3195	3214.1	3192.3	3231.8	2022/9/28		3547.4	3517.4	3587.7
2022/8/22	3340	3241.4	3217.8	3258.7	2022/9/29		3547.5	3517.4	3588.2
2022/8/23	3340	3268.3	3244.2	3286.9	2022/9/30		3547.5	3517.1	3588.2
2022/8/24	3340	3294.9	3268.7	3313.4					
2022/8/25	3340	3321.1	3294.3	3340.8					
2022/8/26	3340	3346.9	3318.5	3367.0					
2022/8/27	3340	3372.4	3342.6	3393.1					

(f) Democratic Republic of the Congo

Date	Observed cumulated cases	Predicted cumulated cases	95% CI		Date	Observed cumulated cases	Predicted cumulated cases	95% CI	
2022/5/8	10	1.5	1.5	1.5	2022/6/15	107	92.4	88.9	96.1
2022/5/9	10	2.1	2.0	2.1	2022/6/16	107	94.4	91.0	98.2
2022/5/10	10	2.6	2.6	2.7	2022/6/17	107	96.3	92.8	99.8
2022/5/11	10	3.3	3.2	3.4	2022/6/18	107	98.2	94.6	101.5
2022/5/12	10	4.0	3.9	4.1	2022/6/19	107	99.9	96.6	103.3
2022/5/13	10	4.8	4.7	4.9	2022/6/20	107	101.5	98.1	104.7
2022/5/14	10	5.6	5.5	5.8	2022/6/21	107	103.0	99.7	106.1
2022/5/15	10	6.5	6.4	6.7	2022/6/22	107	104.4	101.0	107.3
2022/5/16	10	7.5	7.3	7.8	2022/6/23	107	105.7	102.4	108.5
2022/5/17	10	8.6	8.4	8.9	2022/6/24	107	106.9	103.9	109.8
2022/5/18	10	9.8	9.5	10.1	2022/6/25	107	108.1	105.0	110.9
2022/5/19	10	11.1	10.8	11.4	2022/6/26	107	109.2	106.2	112.0
2022/5/20	10	12.5	12.2	12.9	2022/6/27	107	110.2	107.3	113.0
2022/5/21	10	14.0	13.6	14.5	2022/6/28	107	111.2	108.4	114.1
2022/5/22	10	15.7	15.2	16.2	2022/6/29	107	112.1	109.3	114.9
2022/5/23	10	17.5	17.0	18.1	2022/6/30	107	113.0	110.1	115.6
2022/5/24	10	19.5	18.9	20.2	2022/7/1	107	113.8	111.0	116.5
2022/5/25	10	21.7	21.0	22.4	2022/7/2	107	114.5	111.8	117.3
2022/5/26	10	24.0	23.2	24.9	2022/7/3	107	115.2	112.5	118.0
2022/5/27	10	26.6	25.7	27.5	2022/7/4	107	115.9	113.0	118.6
2022/5/28	10	29.4	28.3	30.4	2022/7/5	107	116.5	113.6	119.2
2022/5/29	10	32.4	31.1	33.5	2022/7/6	107	117.1	114.3	119.9
2022/5/30	10	35.7	34.2	37.0	2022/7/7	107	117.7	114.7	120.4
2022/5/31	10	39.3	37.6	40.8	2022/7/8	107	118.2	115.3	121.0
2022/6/1	10	43.2	41.3	45.0	2022/7/9	107	118.7	115.6	121.5
2022/6/2	10	47.5	45.3	49.7	2022/7/10	107	119.1	116.1	122.1
2022/6/3	10	52.2	49.6	54.6	2022/7/11	107	119.6	116.4	122.4
2022/6/4	10	57.2	54.3	60.0	2022/7/12	107	120.0	116.8	122.8
2022/6/5	107	62.8	59.5	66.1	2022/7/13	107	120.3	117.1	123.2
2022/6/6	107	66.6	63.3	70.2	2022/7/14	107	120.7	117.4	123.7
2022/6/7	107	70.3	66.8	74.0	2022/7/15	107	121.0	117.8	124.1
2022/6/8	107	73.7	70.1	77.5	2022/7/16	107	121.4	118.0	124.5
2022/6/9	107	76.9	73.2	80.6	2022/7/17	107	121.6	118.4	124.9
2022/6/10	107	79.9	76.2	83.7	2022/7/18	107	121.9	118.6	125.2
2022/6/11	107	82.7	79.0	86.5	2022/7/19	107	122.2	118.8	125.5
2022/6/12	107	85.3	81.7	89.2	2022/7/20	107	122.4	119.1	125.9
2022/6/13	107	87.8	84.2	91.7	2022/7/21	107	122.7	119.3	126.2
2022/6/14	107	90.2	86.6	94.0	2022/7/22	107	122.9	119.5	126.5

Date	Observed cumulate d cases	Predicted cumulated cases	95% CI		Date	Observed cumulated cases	Predicted cumulated cases	95% CI	
2022/7/23	107	123.1	119.8	126.9	2022/8/27	195	195.4	191.4	199.0
2022/7/24	107	123.3	120.0	127.1	2022/8/28	195	195.4	191.4	199.0
2022/7/25	107	123.5	120.1	127.3	2022/8/29	195	195.4	191.4	199.0
2022/7/26	107	123.6	120.3	127.6	2022/8/30	195	195.4	191.4	199.0
2022/7/27	107	123.8	120.3	127.7	2022/8/31	195	195.4	191.4	199.0
2022/7/28	163	123.9	120.4	127.9	2022/9/1	195	195.4	191.4	199.0
2022/7/29	163	124.4	120.7	128.4	2022/9/2	195	195.4	191.4	199.0
2022/7/30	163	125.1	121.4	129.3	2022/9/3	195	195.4	191.4	199.0
2022/7/31	163	126.2	122.4	130.7	2022/9/4	195	195.4	191.4	199.0
2022/8/1	163	128.0	124.0	132.6	2022/9/5	195	195.4	191.4	199.0
2022/8/2	163	130.7	126.6	135.7	2022/9/6	195	195.4	191.4	199.0
2022/8/3	163	135.0	130.5	140.2	2022/9/7	195	195.4	191.4	199.0
2022/8/4	163	141.7	136.9	146.9	2022/9/8	195	195.4	191.4	199.0
2022/8/5	163	152.3	147.6	157.2	2022/9/9	195	195.4	191.4	199.0
2022/8/6	163	168.9	164.9	173.1	2022/9/10	195	195.4	191.4	199.0
2022/8/7	195	194.9	190.2	199.1	2022/9/11	195	195.4	191.4	199.0
2022/8/8	195	195.0	190.6	198.9	2022/9/12	195	195.4	191.4	199.0
2022/8/9	195	195.2	191.0	198.9	2022/9/13	195	195.4	191.4	199.0
2022/8/10	195	195.2	191.1	198.9	2022/9/14	195	195.4	191.4	199.0
2022/8/11	195	195.3	191.3	199.0	2022/9/15	195	195.4	191.4	199.0
2022/8/12	195	195.3	191.3	198.9	2022/9/16	195	195.4	191.4	199.0
2022/8/13	195	195.4	191.3	198.9	2022/9/17	195	195.4	191.4	199.0
2022/8/14	195	195.4	191.4	199.0	2022/9/18	195	195.4	191.4	199.0
2022/8/15	195	195.4	191.4	199.0	2022/9/19	195	195.4	191.4	199.0
2022/8/16	195	195.4	191.4	199.0	2022/9/20	195	195.4	191.4	199.0
2022/8/17	195	195.4	191.4	199.0	2022/9/21	195	195.4	191.4	199.0
2022/8/18	195	195.4	191.3	199.0	2022/9/22	195	195.4	191.4	199.0
2022/8/19	195	195.4	191.3	199.0	2022/9/23	195	195.4	191.4	199.0
2022/8/20	195	195.4	191.3	199.0	2022/9/24	195	195.4	191.4	199.0
2022/8/21	195	195.4	191.4	199.0	2022/9/25	195	195.4	191.4	199.0
2022/8/22	195	195.4	191.4	199.0	2022/9/26	195	195.4	191.4	199.0
2022/8/23	195	195.4	191.4	199.0	2022/9/27	195	195.4	191.4	199.0
2022/8/24	195	195.4	191.4	199.0	2022/9/28	195	195.4	191.4	199.0
2022/8/25	195	195.4	191.4	199.0	2022/9/29	195	195.4	191.4	199.0
2022/8/26	195	195.4	191.4	199.0	2022/9/30	195	195.4	191.4	199.0

Table S2. The observed cumulated monkeypox cases and simulated cumulated cases by the deterministic SIR model

(a) The global monkeypox cases

Date	Observed cumulated cases	Simulated cumulated cases	Date	Observed cumulated cases	Simulated cumulated cases
2022/1/31	3	1	2022/5/8	37	42
2022/2/16	3	3	2022/5/9	37	46
2022/2/17	6	4	2022/5/10	37	50
2022/2/27	6	5	2022/5/11	37	55
2022/2/28	7	6	2022/5/12	38	61
2022/3/3	7	6	2022/5/13	39	67
2022/3/4	9	7	2022/5/14	39	75
2022/3/30	9	13	2022/5/15	43	84
2022/3/31	15	14	2022/5/16	43	94
2022/4/9	15	17	2022/5/17	46	106
2022/4/10	19	17	2022/5/18	67	120
2022/4/11	19	18	2022/5/19	86	136
2022/4/12	21	18	2022/5/20	131	155
2022/4/13	21	19	2022/5/21	147	176
2022/4/14	21	19	2022/5/22	147	201
2022/4/15	21	19	2022/5/23	220	230
2022/4/16	21	20	2022/5/24	256	263
2022/4/17	21	20	2022/5/25	300	302
2022/4/18	21	21	2022/5/26	388	347
2022/4/19	21	21	2022/5/27	440	399
2022/4/20	21	22	2022/5/28	457	460
2022/4/21	21	22	2022/5/29	477	529
2022/4/22	21	22	2022/5/30	600	611
2022/4/23	21	23	2022/5/31	665	671
2022/4/24	21	23	2022/6/1	749	736
2022/4/25	21	24	2022/6/2	874	805
2022/4/26	21	24	2022/6/3	968	879
2022/4/27	21	25	2022/6/4	976	957
2022/4/28	21	25	2022/6/5	1083	1040
2022/4/29	21	26	2022/6/6	1198	1129
2022/4/30	26	26	2022/6/7	1348	1224
2022/5/1	26	27	2022/6/8	1445	1324
2022/5/2	26	29	2022/6/9	1591	1432
2022/5/3	26	30	2022/6/10	1634	1546
2022/5/4	26	32	2022/6/11	1643	1667
2022/5/5	26	34	2022/6/12	1757	1796
2022/5/6	27	36	2022/6/13	1817	1934
2022/5/7	27	39	2022/6/14	2061	2080

Date	Observed cumulated cases	Simulated cumulated cases	Date	Observed cumulated cases	Simulated cumulated cases
2022/6/15	2208	2235	2022/7/23	17016	17330
2022/6/16	2584	2401	2022/7/24	17050	18006
2022/6/17	2778	2578	2022/7/25	18336	18702
2022/6/18	2783	2765	2022/7/26	19872	19416
2022/6/19	2790	2965	2022/7/27	21025	20150
2022/6/20	3084	3177	2022/7/28	21867	20905
2022/6/21	3366	3403	2022/7/29	23087	21681
2022/6/22	3540	3643	2022/7/30	23172	22480
2022/6/23	4098	3899	2022/7/31	23303	23306
2022/6/24	4303	4172	2022/8/1	24521	24054
2022/6/25	4315	4460	2022/8/2	25963	24824
2022/6/26	4511	4768	2022/8/3	26616	25605
2022/6/27	4898	5096	2022/8/4	27740	26397
2022/6/28	5246	5444	2022/8/5	28907	27199
2022/6/29	5399	5813	2022/8/6	29021	28013
2022/6/30	6206	6208	2022/8/7	29193	28838
2022/7/1	6475	6545	2022/8/8	31178	29674
2022/7/2	6519	6895	2022/8/9	32613	30521
2022/7/3	6559	7258	2022/8/10	33766	31379
2022/7/4	7053	7631	2022/8/11	34451	32248
2022/7/5	7500	8017	2022/8/12	35863	33128
2022/7/6	7732	8413	2022/8/13	36004	34020
2022/7/7	8426	8821	2022/8/14	36172	34923
2022/7/8	9486	9243	2022/8/15	37646	35838
2022/7/9	9608	9678	2022/8/16	38965	36763
2022/7/10	9635	10126	2022/8/17	40147	37700
2022/7/11	10226	10588	2022/8/18	41192	38647
2022/7/12	11098	11064	2022/8/19	41964	39606
2022/7/13	11516	11552	2022/8/20	42130	40577
2022/7/14	12127	12056	2022/8/21	42238	41558
2022/7/15	13015	12576	2022/8/22	44301	42551
2022/7/16	13031	13112	2022/8/23	45752	43554
2022/7/17	13069	13664	2022/8/24	46801	44569
2022/7/18	13886	14232	2022/8/25	47438	45595
2022/7/19	15081	14816	2022/8/26	48504	46633
2022/7/20	15655	15416	2022/8/27	48563	47681
2022/7/21	16293	16035	2022/8/28	48616	48740
2022/7/22	17013	16673	2022/8/29	50343	49810

Date	Observed cumulated cases	Simulated cumulated cases	Date	Observed cumulated cases	Simulated cumulated cases
2022/8/30	51110	50891			
2022/8/31	51982	51988			
2022/9/1	52941	52744			
2022/9/2	53629	53490			
2022/9/3	53678	54216			
2022/9/4	53936	54920			
2022/9/5	54939	55606			
2022/9/6	56138	56274			
2022/9/7	56852	56925			
2022/9/8	57490	57560			
2022/9/9	58211	58177			
2022/9/10	58322	58778			
2022/9/11	58392	59362			
2022/9/12	59351	59928			
2022/9/13	60337	60477			
2022/9/14	60784	61011			
2022/9/15	61581	61531			
2022/9/16	62324	62038			
2022/9/17	62399	62531			
2022/9/18	62403	63012			
2022/9/19	63758	63479			
2022/9/20	64418	63933			
2022/9/21	64904	64373			
2022/9/22	65215	64800			
2022/9/23	65215	65214			

(b) **United States**

Date	Observed cumulated cases	Simulated cumulated cases	Date	Observed cumulated cases	Simulated cumulated cases
2022/5/18	1	1.0	2022/6/25	243	247.7
2022/5/19	2	1.3	2022/6/26	252	281.6
2022/5/20	2	1.6	2022/6/27	315	320.2
2022/5/21	2	1.9	2022/6/28	359	364.0
2022/5/22	2	2.3	2022/6/29	409	413.8
2022/5/23	2	2.7	2022/6/30	470	470.5
2022/5/24	4	3.2	2022/7/1	543	517.0
2022/5/25	6	3.8	2022/7/2	547	567.0
2022/5/26	11	4.4	2022/7/3	553	620.0
2022/5/27	15	5.2	2022/7/4	563	677.0
2022/5/28	16	6.0	2022/7/5	709	739.0
2022/5/29	17	6.9	2022/7/6	749	804.0
2022/5/30	17	8.0	2022/7/7	883	874.0
2022/5/31	21	9.2	2022/7/8	950	949.0
2022/6/1	22	10.6	2022/7/9	960	1028.0
2022/6/2	26	12.2	2022/7/10	964	1114.0
2022/6/3	31	14.0	2022/7/11	1058	1205.0
2022/6/4	32	16.0	2022/7/12	1122	1303.0
2022/6/5	32	18.3	2022/7/13	1245	1407.0
2022/6/6	34	20.9	2022/7/14	1426	1519.0
2022/6/7	40	23.9	2022/7/15	1767	1638.0
2022/6/8	45	27.3	2022/7/16	1767	1766.0
2022/6/9	50	31.1	2022/7/17	1767	1903.0
2022/6/10	53	35.5	2022/7/18	1995	2049.0
2022/6/11	55	40.5	2022/7/19	2114	2204.0
2022/6/12	57	46.1	2022/7/20	2323	2371.0
2022/6/13	75	52.6	2022/7/21	2503	2550.0
2022/6/14	83	59.9	2022/7/22	2875	2740.0
2022/6/15	96	68.2	2022/7/23	2875	2944.0
2022/6/16	117	77.6	2022/7/24	2875	3163.0
2022/6/17	128	88.3	2022/7/25	3457	3396.0
2022/6/18	132	100.5	2022/7/26	3768	3645.0
2022/6/19	133	114.4	2022/7/27	4630	3912.0
2022/6/20	137	130.1	2022/7/28	4895	4197.0
2022/6/21	175	148.0	2022/7/29	5175	4501.0
2022/6/22	196	168.4	2022/7/30	5175	4826.0
2022/6/23	218	191.5	2022/7/31	5175	5174.0
2022/6/24	237	217.8	2022/8/1	5792	5466.0

Date	Observed cumulated cases	Simulated cumulated cases	Date	Observed cumulated cases	Simulated cumulated cases
2022/8/2	6308	5760.0	2022/9/9	21761	21626.0
2022/8/3	6599	6062.0	2022/9/10	21761	21876.0
2022/8/4	7084	6372.0	2022/9/11	21761	22118.0
2022/8/5	7490	6691.0	2022/9/12	21834	22351.0
2022/8/6	7491	7018.0	2022/9/13	22480	22575.0
2022/8/7	7491	7355.0	2022/9/14	22619	22791.0
2022/8/8	8902	7702.0	2022/9/15	22958	22999.0
2022/8/9	9461	8058.0	2022/9/16	23339	23198.0
2022/8/10	10361	8423.0	2022/9/17	23339	23390.0
2022/8/11	10727	8798.0	2022/9/18	23339	23575.0
2022/8/12	11131	9183.0	2022/9/19	23730	23754.0
2022/8/13	11131	9578.0	2022/9/20	24041	23926.0
2022/8/14	11131	9985.0	2022/9/21	24198	24091.0
2022/8/15	11844	10404.0	2022/9/22	24403	24250.0
2022/8/16	12636	10834.0	2022/9/23	24403	24402.0
2022/8/17	13450	11276.0	2022/9/9	21761	21626.0
2022/8/18	14049	11729.0	2022/9/10	21761	21876.0
2022/8/19	14049	12193.0	2022/9/11	21761	22118.0
2022/8/20	14049	12670.0	2022/9/12	21834	22351.0
2022/8/21	14049	13162.0	2022/9/13	22480	22575.0
2022/8/22	15357	13668.0	2022/9/14	22619	22791.0
2022/8/23	15832	14187.0	2022/9/15	22958	22999.0
2022/8/24	16514	14721.0	2022/9/16	23339	23198.0
2022/8/25	16837	15268.0	2022/9/17	23339	23390.0
2022/8/26	17336	15830.0	2022/9/18	23339	23575.0
2022/8/27	17336	16407.0	2022/9/19	23730	23754.0
2022/8/28	17336	16999.0	2022/9/20	24041	23926.0
2022/8/29	17986	17608.0	2022/9/21	24198	24091.0
2022/8/30	18298	18233.0	2022/9/22	24403	24250.0
2022/8/31	18879	18876.0	2022/9/23	24403	24402.0
2022/9/1	19355	19236.0			
2022/9/2	19852	19575.0			
2022/9/3	19852	19902.0			
2022/9/4	19852	20216.0			
2022/9/5	19852	20520.0			
2022/9/6	20608	20813.0			
2022/9/7	21148	21095.0			
2022/9/8	21374	21366.0			

(c) **Spain**

Date	Observed cumulated cases	Simulated cumulated cases	Date	Observed cumulated cases	Simulated cumulated cases
2022/5/18	7	7.0	2022/6/25	736	919.4
2022/5/19	7	8.8	2022/6/26	736	975.2
2022/5/20	30	10.8	2022/6/27	800	1032.9
2022/5/21	40	13.2	2022/6/28	800	1092.5
2022/5/22	40	15.8	2022/6/29	800	1154.4
2022/5/23	41	18.8	2022/6/30	1196	1218.6
2022/5/24	45	22.1	2022/7/1	1196	1285.1
2022/5/25	53	25.9	2022/7/2	1196	1354.0
2022/5/26	78	30.2	2022/7/3	1196	1425.2
2022/5/27	99	35.1	2022/7/4	1258	1498.9
2022/5/28	99	40.7	2022/7/5	1258	1575.5
2022/5/29	108	46.9	2022/7/6	1258	1655.0
2022/5/30	116	54.1	2022/7/7	1258	1737.2
2022/5/31	136	62.1	2022/7/8	2034	1822.3
2022/6/1	136	71.2	2022/7/9	2034	1910.1
2022/6/2	162	81.6	2022/7/10	2034	2001.4
2022/6/3	181	93.2	2022/7/11	2034	2096.0
2022/6/4	181	106.5	2022/7/12	2447	2194.2
2022/6/5	181	121.5	2022/7/13	2447	2295.8
2022/6/6	198	138.5	2022/7/14	2447	2400.8
2022/6/7	225	157.8	2022/7/15	2835	2509.5
2022/6/8	259	179.6	2022/7/16	2835	2622.0
2022/6/9	259	204.3	2022/7/17	2835	2711.7
2022/6/10	275	232.3	2022/7/18	2835	2799.8
2022/6/11	275	264.0	2022/7/19	3125	2887.9
2022/6/12	275	299.8	2022/7/20	3125	2975.9
2022/6/13	275	340.5	2022/7/21	3125	3063.9
2022/6/14	313	386.5	2022/7/22	3126	3151.9
2022/6/15	313	438.7	2022/7/23	3126	3239.8
2022/6/16	497	497.8	2022/7/24	3126	3327.7
2022/6/17	498	538.5	2022/7/25	3126	3415.6
2022/6/18	498	580.4	2022/7/26	3738	3503.4
2022/6/19	498	623.8	2022/7/27	3738	3591.1
2022/6/20	521	668.8	2022/7/28	3738	3678.8
2022/6/21	521	715.5	2022/7/29	4299	3766.5
2022/6/22	521	763.8	2022/7/30	4300	3854.1
2022/6/23	736	813.8	2022/7/31	4300	3941.7
2022/6/24	736	865.6	2022/8/1	4300	4029.2

Date	Observed cumulated cases	Simulated cumulated cases	Date	Observed cumulated cases	Simulated cumulated cases
2022/8/2	4577	4116.7	2022/9/9	6884	6890.4
2022/8/3	4577	4204.1	2022/9/10	6884	6910.8
2022/8/4	4577	4291.5	2022/9/11	6884	6929.9
2022/8/5	4942	4378.9	2022/9/12	6884	6947.9
2022/8/6	4942	4466.2	2022/9/13	6947	6964.7
2022/8/7	4942	4553.4	2022/9/14	6947	6980.4
2022/8/8	4942	4640.6	2022/9/15	6947	6995.1
2022/8/9	5162	4727.8	2022/9/16	7037	7009.0
2022/8/10	5162	4814.8	2022/9/17	7037	7021.9
2022/8/11	5162	4901.8	2022/9/18	7037	7033.9
2022/8/12	5719	4988.8	2022/9/19	7037	7045.2
2022/8/13	5719	5075.7	2022/9/20	7083	7055.9
2022/8/14	5719	5162.6	2022/9/21	7083	7065.8
2022/8/15	5719	5249.4	2022/9/22	7083	7075.0
2022/8/16	5792	5336.1	2022/9/23	7083	7083.7
2022/8/17	5792	5422.8	2022/9/9	6884	6890.4
2022/8/18	5792	5509.4	2022/9/10	6884	6910.8
2022/8/19	6119	5596.0	2022/9/11	6884	6929.9
2022/8/20	6119	5682.5	2022/9/12	6884	6947.9
2022/8/21	6119	5768.9	2022/9/13	6947	6964.7
2022/8/22	6119	5855.3	2022/9/14	6947	6980.4
2022/8/23	6284	5941.6	2022/9/15	6947	6995.1
2022/8/24	6284	6027.8	2022/9/16	7037	7009.0
2022/8/25	6284	6114.0	2022/9/17	7037	7021.9
2022/8/26	6459	6200.0	2022/9/18	7037	7033.9
2022/8/27	6459	6286.1	2022/9/19	7037	7045.2
2022/8/28	6459	6372.0	2022/9/20	7083	7055.9
2022/8/29	6459	6457.9	2022/9/21	7083	7065.8
2022/8/30	6543	6543.8	2022/9/22	7083	7075.0
2022/8/31	6543	6629.5	2022/9/23	7083	7083.7
2022/9/1	6543	6667.7			
2022/9/2	6645	6702.3			
2022/9/3	6645	6734.6			
2022/9/4	6645	6765.0			
2022/9/5	6645	6793.6			
2022/9/6	6749	6820.2			
2022/9/7	6749	6845.1			
2022/9/8	6749	6868.4			

(d) **Brazil**

Date	Observed cumulated cases	Simulated cumulated cases	Date	Observed cumulated cases	Simulated cumulated cases
2022/6/8	1	1	2022/7/16	347	407.9
2022/6/9	1	1.3	2022/7/17	347	444.6
2022/6/10	1	1.6	2022/7/18	347	483.8
2022/6/11	2	2.1	2022/7/19	448	526
2022/6/12	3	2.5	2022/7/20	591	571.3
2022/6/13	3	3.1	2022/7/21	604	619.7
2022/6/14	5	3.8	2022/7/22	694	671.9
2022/6/15	5	4.5	2022/7/23	694	727.9
2022/6/16	6	5.5	2022/7/24	694	787.8
2022/6/17	7	6.5	2022/7/25	809	852.2
2022/6/18	7	7.8	2022/7/26	865	921.4
2022/6/19	8	9.2	2022/7/27	977	995.4
2022/6/20	8	10.9	2022/7/28	1066	1075
2022/6/21	9	13	2022/7/29	1259	1160.5
2022/6/22	11	15.3	2022/7/30	1343	1251.8
2022/6/23	16	18.1	2022/7/31	1370	1350.1
2022/6/24	17	21.3	2022/8/1	1475	1455.8
2022/6/25	19	25.1	2022/8/2	1603	1568.6
2022/6/26	20	29.5	2022/8/3	1721	1689.9
2022/6/27	20	34.7	2022/8/4	1860	1820.3
2022/6/28	21	40.8	2022/8/5	2004	1959.9
2022/6/29	37	48	2022/8/6	2108	2109.7
2022/6/30	49	56.4	2022/8/7	2131	2214.2
2022/7/1	64	66.2	2022/8/8	2293	2320.1
2022/7/2	76	77.7	2022/8/9	2415	2426.9
2022/7/3	78	91.2	2022/8/10	2458	2534.8
2022/7/4	80	107	2022/8/11	2458	2643.6
2022/7/5	106	125.5	2022/8/12	2746	2753.5
2022/7/6	142	147.2	2022/8/13	2848	2864.3
2022/7/7	172	172.7	2022/8/14	2893	2976.2
2022/7/8	204	191.9	2022/8/15	2985	3089.1
2022/7/9	218	212.7	2022/8/16	3183	3203
2022/7/10	218	235	2022/8/17	3359	3317.9
2022/7/11	228	259	2022/8/18	3450	3433.8
2022/7/12	266	284.7	2022/8/19	3655	3550.8
2022/7/13	308	312.3	2022/8/20	3755	3668.7
2022/7/14	347	342	2022/8/21	3787	3787.7
2022/7/15	347	373.7	2022/8/22	3896	3907.7

Date	Observed cumulated cases	Simulated cumulated cases	Date	Observed cumulated cases	Simulated cumulated cases
2022/8/23	3984	4028.9			
2022/8/24	4144	4151.2			
2022/8/25	4216	4274.6			
2022/8/26	4472	4399.1			
2022/8/27	4472	4524.6			
2022/8/28	4472	4651.3			
2022/8/29	4692	4779			
2022/8/30	4876	4907.9			
2022/8/31	5037	5037.8			
2022/9/1	5197	5150.4			
2022/9/2	5197	5259.4			
2022/9/3	5197	5367.4			
2022/9/4	5409	5474.2			
2022/9/5	5525	5579.9			
2022/9/6	5692	5684.4			
2022/9/7	5726	5787.9			
2022/9/8	5852	5890.2			
2022/9/9	5971	5991.4			
2022/9/10	6014	6091.6			
2022/9/11	6032	6190.7			
2022/9/12	6129	6288.7			
2022/9/13	6246	6385.8			
2022/9/14	6448	6481.9			
2022/9/15	6649	6576.9			
2022/9/16	6806	6670.9			
2022/9/17	6867	6763.9			
2022/9/18	6867	6855.9			
2022/9/19	7018	6946.9			
2022/9/20	7115	7036.9			
2022/9/21	7205	7125.8			
2022/9/22	7300	7213.9			
2022/9/23	7300	7301			

(e) **United Kingdom**

Date	Observed cumulated cases	Simulated cumulated cases	Date	Observed cumulated cases	Simulated cumulated cases
2022/5/6	1	1.0	2022/6/13	470	445.4
2022/5/7	1	1.3	2022/6/14	524	474.6
2022/5/8	1	1.7	2022/6/15	524	504.8
2022/5/9	1	2.1	2022/6/16	574	536.2
2022/5/10	1	2.6	2022/6/17	574	568.9
2022/5/11	1	3.2	2022/6/18	574	602.7
2022/5/12	2	4.0	2022/6/19	574	637.7
2022/5/13	3	4.8	2022/6/20	793	674.0
2022/5/14	3	5.8	2022/6/21	793	711.7
2022/5/15	7	7.0	2022/6/22	793	750.9
2022/5/16	7	8.5	2022/6/23	910	791.6
2022/5/17	7	10.2	2022/6/24	910	833.6
2022/5/18	9	12.2	2022/6/25	910	877.2
2022/5/19	9	14.5	2022/6/26	1076	922.5
2022/5/20	20	17.3	2022/6/27	1076	969.6
2022/5/21	20	20.6	2022/6/28	1076	1018.4
2022/5/22	20	24.6	2022/6/29	1076	1069.0
2022/5/23	57	29.2	2022/6/30	1235	1121.3
2022/5/24	71	34.7	2022/7/1	1235	1175.6
2022/5/25	78	41.2	2022/7/2	1235	1232.0
2022/5/26	106	48.9	2022/7/3	1235	1290.4
2022/5/27	106	58.0	2022/7/4	1351	1351.4
2022/5/28	106	68.8	2022/7/5	1351	1395.5
2022/5/29	106	81.6	2022/7/6	1351	1440.5
2022/5/30	179	96.8	2022/7/7	1552	1485.2
2022/5/31	190	114.7	2022/7/8	1552	1529.8
2022/6/1	196	135.9	2022/7/9	1552	1574.2
2022/6/2	207	161.0	2022/7/10	1552	1618.3
2022/6/3	226	190.7	2022/7/11	1735	1662.3
2022/6/4	226	226.0	2022/7/12	1735	1706.1
2022/6/5	226	246.8	2022/7/13	1735	1749.7
2022/6/6	302	268.5	2022/7/14	1856	1793.0
2022/6/7	321	291.1	2022/7/15	1856	1836.2
2022/6/8	321	314.6	2022/7/16	1856	1879.3
2022/6/9	366	338.8	2022/7/17	1856	1922.1
2022/6/10	366	364.0	2022/7/18	2137	1964.7
2022/6/11	366	390.1	2022/7/19	2137	2007.1
2022/6/12	470	417.3	2022/7/20	2137	2049.3

Date	Observed cumulated cases	Simulated cumulated cases	Date	Observed cumulated cases	Simulated cumulated cases
2022/7/21	2208	2091.4	2022/8/28	3340	3428.3
2022/7/22	2208	2133.2	2022/8/29	3413	3440.0
2022/7/23	2208	2174.9	2022/8/30	3413	3451.0
2022/7/24	2208	2216.3	2022/8/31	3413	3461.4
2022/7/25	2497	2257.6	2022/9/1	3413	3471.2
2022/7/26	2497	2298.7	2022/9/2	3413	3480.3
2022/7/27	2497	2339.7	2022/9/3	3413	3488.9
2022/7/28	2546	2380.4	2022/9/4	3413	3497.0
2022/7/29	2546	2420.9	2022/9/5	3484	3504.6
2022/7/30	2546	2461.3	2022/9/6	3484	3511.7
2022/7/31	2546	2501.5	2022/9/7	3484	3518.4
2022/8/1	2759	2541.5	2022/9/8	3484	3524.7
2022/8/2	2759	2581.3	2022/9/9	3484	3530.6
2022/8/3	2759	2620.9	2022/9/10	3484	3536.2
2022/8/4	2859	2660.4	2022/9/11	3484	3541.4
2022/8/5	2859	2699.7	2022/9/12	3552	3546.3
2022/8/6	2859	2738.7	2022/9/13	3552	3550.9
2022/8/7	2859	2777.6	2022/9/14	3552	3555.2
2022/8/8	3017	2816.4	2022/9/15	3552	3559.2
2022/8/9	3017	2854.9	2022/9/16	3552	3563.0
2022/8/10	3017	2893.3	2022/9/17	3552	3566.6
2022/8/11	3017	2931.5	2022/9/18	3552	3570.0
2022/8/12	3017	2969.5	2022/9/19	3552	3573.1
2022/8/13	3017	3007.4	2022/9/20	3585	3576.1
2022/8/14	3017	3045.1	2022/9/21	3585	3578.9
2022/8/15	3195	3082.6	2022/9/22	3585	3581.5
2022/8/16	3195	3119.9	2022/9/23	3585	3583.9
2022/8/17	3195	3157.1			
2022/8/18	3195	3194.1			
2022/8/19	3195	3230.9			
2022/8/20	3195	3267.5			
2022/8/21	3195	3304.0			
2022/8/22	3340	3340.6			
2022/8/23	3340	3357.0			
2022/8/24	3340	3373.1			
2022/8/25	3340	3388.1			
2022/8/26	3340	3402.3			
2022/8/27	3340	3415.7			

(f) **Democratic Republic of the Congo**

Date	Observed cumulated cases	Simulated cumulated cases	Date	Observed cumulated cases	Simulated cumulated cases
2022/5/8	10	10.0	2022/6/15	107	133.4
2022/5/9	10	11.7	2022/6/16	107	135.1
2022/5/10	10	13.5	2022/6/17	107	136.7
2022/5/11	10	15.3	2022/6/18	107	138.2
2022/5/12	10	17.3	2022/6/19	107	139.6
2022/5/13	10	19.4	2022/6/20	107	140.9
2022/5/14	10	21.5	2022/6/21	107	142.2
2022/5/15	10	23.8	2022/6/22	107	143.4
2022/5/16	10	26.1	2022/6/23	107	144.6
2022/5/17	10	28.6	2022/6/24	107	145.7
2022/5/18	10	31.2	2022/6/25	107	146.7
2022/5/19	10	34.0	2022/6/26	107	147.7
2022/5/20	10	36.9	2022/6/27	107	148.7
2022/5/21	10	39.9	2022/6/28	107	149.6
2022/5/22	10	43.0	2022/6/29	107	150.4
2022/5/23	10	46.3	2022/6/30	107	151.2
2022/5/24	10	49.8	2022/7/1	107	152.0
2022/5/25	10	53.5	2022/7/2	107	152.7
2022/5/26	10	57.3	2022/7/3	107	153.4
2022/5/27	10	61.3	2022/7/4	107	154.1
2022/5/28	10	65.6	2022/7/5	107	154.7
2022/5/29	10	70.0	2022/7/6	107	155.3
2022/5/30	10	74.6	2022/7/7	107	155.9
2022/5/31	10	79.5	2022/7/8	107	156.4
2022/6/1	10	84.6	2022/7/9	107	156.9
2022/6/2	10	90.0	2022/7/10	107	157.4
2022/6/3	10	95.6	2022/7/11	107	157.9
2022/6/4	10	101.5	2022/7/12	107	158.3
2022/6/5	107	107.6	2022/7/13	107	158.7
2022/6/6	107	114.2	2022/7/14	107	159.1
2022/6/7	107	116.7	2022/7/15	107	159.5
2022/6/8	107	119.2	2022/7/16	107	159.9
2022/6/9	107	121.5	2022/7/17	107	160.2
2022/6/10	107	123.8	2022/7/18	107	160.5
2022/6/11	107	125.9	2022/7/19	107	160.8
2022/6/12	107	128.0	2022/7/20	107	161.1
2022/6/13	107	129.9	2022/7/21	107	161.4
2022/6/14	107	131.7	2022/7/22	107	161.7

Date	Observed cumulated cases	Simulated cumulated cases	Date	Observed cumulated cases	Simulated cumulated cases
2022/7/23	107	161.9	2022/8/20	195	-
2022/7/24	107	162.2	2022/8/21	195	-
2022/7/25	107	162.4	2022/8/22	195	-
2022/7/26	107	162.6	2022/8/23	195	-
2022/7/27	107	162.8	2022/8/24	195	-
2022/7/28	163	163.0	2022/8/25	195	-
2022/7/29	163	164.0	2022/8/26	195	-
2022/7/30	163	165.3	2022/8/27	195	-
2022/7/31	163	166.9	2022/8/28	195	-
2022/8/1	163	168.9	2022/8/29	195	-
2022/8/2	163	171.3	2022/8/30	195	-
2022/8/3	163	174.3	2022/8/31	195	-
2022/8/4	163	178.0	2022/9/1	195	-
2022/8/5	163	182.6	2022/9/2	195	-
2022/8/6	163	188.2	2022/9/3	195	-
2022/8/7	195	195.2	2022/9/4	195	-
2022/8/8	195	-	2022/9/5	195	-
2022/8/9	195	-	2022/9/6	195	-
2022/8/10	195	-	2022/9/7	195	-
2022/8/11	195	-	2022/9/8	195	-
2022/8/12	195	-	2022/9/9	195	-
2022/8/13	195	-	2022/9/10	195	-
2022/8/14	195	-	2022/9/11	195	-
2022/8/15	195	-	2022/9/12	195	-
2022/8/16	195	-	2022/9/13	195	-
2022/8/17	195	-	2022/9/14	195	-
2022/8/18	195	-	2022/9/15	195	-
2022/8/19	195	-	2022/9/16	195	-
